# Test for Blood

**Published:** 1890-01

**Authors:** 


### Test for Blood.

A simple test for blood, and one easy of application, is made by the addition of tincture of guaiac and ozonized ether to a weak solution of blood, when a bright blue coloration is produced. If a drop of blood be mixed with half an ounce of distilled water, upon the addition of one or two drops of tincture of guaiac, a cloudy precipitate of the resin appears, and the solution has a faint tint. If to this solution one drop of an ethereal solution of hydrogen peroxide is added a blue tint appears, which, upon a few minutes exposure, gradually deepens. This test is very valuable for minute quantities of blood, and one experimentor has succeeded in obtaining impressions from a stain upon cloth where the microscope failed to show any blood.—*Druggists' Bulletin.*

THE following is a broad rule: Dropsy of the feet alone means heart, dropsy of the belly alone means liver, and dropsy of all the body means kidneys.—*Med. Summary.*

